# Monitoring Long-Term Vegetation Dynamics in the Hulun Lake Basin of Northeastern China Through Greening and Browning Speeds from 1982 to 2015

**DOI:** 10.3390/plants14213394

**Published:** 2025-11-05

**Authors:** Nan Shan, Tie Wang, Qian Zhang, Jinqi Gong, Mingzhu He, Xiaokang Zhang, Xuehe Lu, Feng Qiu

**Affiliations:** 1 Nanjing lnstitute of Environmental Sciences, Ministry of Ecology and Environment of the People’s Republic of China, Nanjing 210042, China; shan.nan@nies.org (N.S.); wtwt@njtech.edu.cn (T.W.);; 2 Inner Mongolia Hulun Lake (Wetland) Comprehensive Monitoring Station for Ecological Quality, Hulunbuir 021000, China; 3 School of Geomatics Science and Technology, Nanjing Tech University, Nanjing 211816, China; 4 College of Geodesy and Geomatics, Shandong University of Science and Technology, Qingdao 266590, China; zhangxiaokang@sdust.edu.cn; 5 Chinese Academy of Surveying & Mapping, Beijing 100036, China; 6 School of National Safety and Emergency Management, Beijing Normal University, Zhuhai 519087, China; hemingzhu@bnu.edu.cn; 7 School of Geography Science and Geomatics Engineering, Suzhou University of Science and Technology, Suzhou 215000, China

**Keywords:** vegetation dynamics, greening and browning speed, VNDVI, process-level changes

## Abstract

Vegetation dynamics in the Hulun Lake Basin (HLB), a vulnerable grassland–wetland–forest transition zone in Northeastern Inner Mongolia, North China, are sensitive to climate change, but traditional greenness metrics like the normalized difference vegetation index (NDVI) lack process-level insights. Using the GIMMS NDVI3g dataset (1982–2015) and meteorological data, this study analyzed the spatiotemporal dynamics of the NDVI and vegetation NDVI change rate (VNDVI)—a metric quantifying greening and browning speeds via NDVI temporal variation—employing linear regression and partial correlation analyses. The NDVI exhibited an overall significant upward trend of +0.0028 yr^−1^ (*p* < 0.05) across more than 70% of the basin, indicating a persistent greening tendency. The VNDVI revealed an accelerated spring greening rate of +0.8% yr^−1^ (*p* < 0.05) and a slowed autumn browning rate of −0.6% yr^−1^ (*p* < 0.05), reflecting an extended growing season. Spatial correlation analysis showed that the temperature dominated spring greening (r = 0.52), precipitation governed summer growth (r = 0.64), and solar radiation modulated autumn senescence (r = 0.38). Compared with the NDVI, the VNDVI was more sensitive to both climatic fluctuations and anthropogenic disturbances, highlighting its utility in capturing process-level vegetation dynamics. These findings provide quantitative insights into the mechanisms of vegetation change in the HLB and offer scientific support for ecological conservation in North China’s grassland–forest ecotone.

## 1. Introduction

Vegetation is a key component of terrestrial ecosystems, providing critical ecological functions such as climate regulation, biodiversity support, and the maintenance of essential ecosystem services [1]. To adapt to the seasonal and periodic changes in the environment, vegetation has also developed growth and development rhythms and cycles [2]. Vegetation phenology is employed to study the patterns of recurring biological events in plants and their relationships with environmental factors such as the climate, season, and habitat conditions [3]. In temperate regions, due to the environmental stress of low temperatures in winter and the favorable growth conditions in summer, vegetation exhibits distinct seasonal changes within a year: from germination, growth, flowering, and fruiting to leaf senescence and abscission [4]. In arid and semi-arid regions, vegetation phenological dynamics are particularly sensitive to environmental changes, making them effective indicators of both climatic variability and anthropogenic disturbances [5].

With the increasing pace of global climate change, analyzing the spatiotemporal characteristics of vegetation growth has become essential for ecological monitoring and sustainable land management [6]. Such analysis contributes to understanding ecosystem responses and resilience under multiple stressors [7]. Furthermore, vegetation also exerts important feedback on the entire Earth system through interactions among soil, air, and water across multiple spheres [8]. Therefore, understanding the drivers of plant dynamic changes is crucial in predicting the impacts of future climate warming on the carbon cycle and the climate feedback of terrestrial ecosystems [9].

Vegetation phenology is a fundamental ecological indicator in assessing ecosystem functioning [10]. The start of season (SOS) refers to the time when vegetation begins to show signs of growth or greening at the start of the growing season. The peak of season (POS) refers to the period when vegetation reaches its maximum growth or greenness during the growing season. The end of season (EOS) marks the time when vegetation starts to decline or lose its greenness, indicating the end of the growing season. Phenological parameters, such as the start and end of the growing season, reflecting the seasonal timing of plant activity, have been well studied in the past few decades with advances in multi-scale remote sensing observations [11]. Based on the widely used normalized difference vegetation index (NDVI), many studies reveal advancing spring green-up (SOS) and delayed autumn senescence (EOS), and therefore extending growing seasons, primarily driven by warming and CO_2_ fertilization [12]. High-resolution satellite data (e.g., Sentinel-2, PlanetScope) combined with solar-induced chlorophyll fluorescence (SIF) enable the near-real-time monitoring of photosynthetic phenology, outperforming traditional NDVI-based methods [13]. Through climate manipulation experiments at a temperate forest site, Zani et al. [14] revealed that, differing from delays in autumn senescence dates, slight advances might occur due to the limitation of plant carbon uptake during the growing season imposing strong constraints on the length of the productive season through feedback between source and sink organs in plants depending on the environmental conditions [15]. Meanwhile, another study also demonstrated that intrinsic biological controls, such as the nearly stable proportions of time allocated to greening over browning, buffer northern ecosystems against climate variability through SOS→POS→EOS carryover effects. However, extreme climates may alter this constant time allocation, highlighting the need for further understanding of the responses of phenological processes (including both greening and browning) to varying climates [16]. Greening and browning speeds, quantified as the rates of change in phenological dynamics between consecutive periods, usually in the form of the velocity of NDVI variation (VNDVI), offer a mechanistic alternative for the monitoring of vegetation activity; this is different from the greenness NDVI itself [17]. The NDVI represents the magnitude of canopy greenness, while the VNDVI is the speed of canopy greening and browning [18]. The greening speed (GRS, with a positive VNDVI) reflects the acceleration of canopy development, linked to carbon allocation to leaves and mainly affected by the environment, while the browning speed (BRS, with a negative VNDVI) reveals senescence kinetics, modulated by climate conditions and sink–source relationships [19]. In temperate grassland systems with strong seasonality and ecological vulnerability, both indicators exhibit high sensitivity to temperature and precipitation changes, making them valuable tools in detecting ecosystem change [20].

The Hulun Lake Basin (HLB), located in Hulunbuir City in the Inner Mongolia Autonomous Region of China, represents a key grassland–wetland–forest ecotone on the eastern edge of the Mongolian Plateau, serving as an important ecological barrier in North China. It is characterized by high climatic sensitivity and strong spatial heterogeneity, making it an ideal region for the investigation of vegetation dynamics and their responses to environmental change [7,21]. Over the past few decades, the HLB has undergone significant warming and drying, accompanied by intensified anthropogenic pressures such as overgrazing, agricultural expansion, and urbanization [8,10,11]. These combined climatic and human influences have led to the notable degradation of the grassland and wetland ecosystems, particularly around Hulun Lake itself [6]. Therefore, monitoring the vegetation dynamics in this transboundary basin between China and Mongolia is essential in understanding ecological change processes and informing regional conservation and management practices [6,9].

This study aims to address these gaps by analyzing the spatiotemporal patterns of vegetation canopy greening and browning speeds in the HLB from 1982 to 2015, based on long-term satellite-derived NDVI time series [22]. We examine spatiotemporal trends in the NDVI and VNDVI to reveal the spatial heterogeneity and temporal evolution of vegetation phenological dynamics. Additionally, the relationships between the canopy greening/browning speeds and key climatic variables (e.g., temperature, precipitation, and solar radiation) are investigated to identify the dominant climatic drivers of vegetation change.

## 2. Results

### 2.1. Variations in Vegetation Greenness

**Figure 1:**
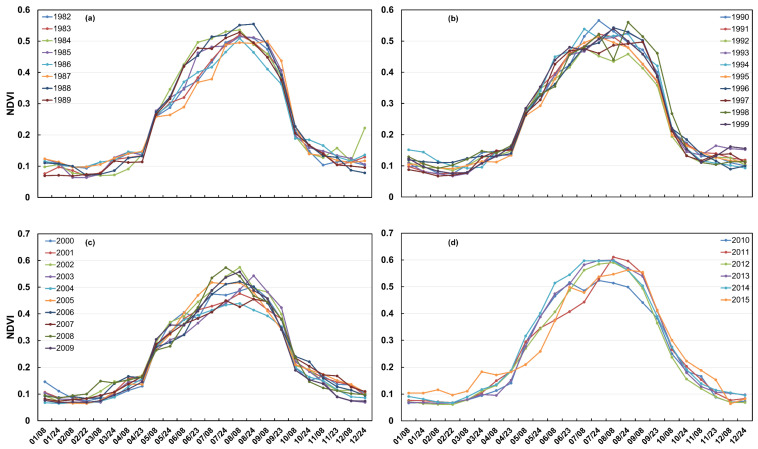
(**a**) The trends of the annual and biweekly NDVI changes in the HLB from 1982 to 1989, (**b**) The trends of the annual and biweekly NDVI changes in the HLB from 1990 to 1999, (**c**) The trends of the annual and biweekly NDVI changes in the HLB from 2000 to 2009, (**d**) The trends of the annual and biweekly NDVI changes in the HLB from 2010 to 2015.

**Figure 2:**
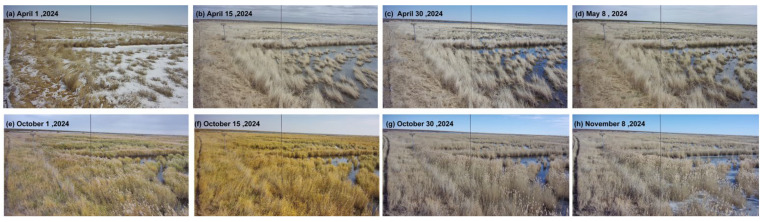
Images of the grassland near the Kherlen River in the central HLB during the growing season in 2024.

Changes in vegetation phenology serve as critical indicators of terrestrial ecosystem functioning. Figure 1 illustrates the intra-annual biweekly variations in the NDVI across four distinct periods within the HLB: three full decades (1982–1989, 1990–1999, 2000–2009) and a shorter final period (2010–2015). The seasonal cycles showed that the NDVI peak time varied between July (accounting for 60%) and August (accounting for 30%) throughout the study period from 1982 to 2015 and more recently shifted to July after 2000. From the perspective of the annual growth curves, the regional NDVI begins to increase around March and shows a pronounced rise in April. Between August and September, the NDVI remains relatively stable, with only minor changes, while a decreasing trend becomes evident in October. However, the NDVI values remain relatively low (approximately 0.2–0.3) at the beginning of April. Snow cover often persists in the grasslands surrounding the HLB during early spring. The increase in the NDVI from March to May is largely attributed to snowmelt, which significantly reduces reflectance in the red band (Figure 2). Similarly, the sharp decline in the NDVI observed from October to November is also influenced by the onset of snowfall. Based on these patterns, May is defined as the beginning of the growing season, and October is designated as its end, which has been widely adopted in previous studies. Accordingly, the vegetation canopy greening stage is defined as the period from May to early August, while the browning stage extends from late August to November. In the following analyses, we still include April and November to better understand changes in the surrounding climate.

**Figure 3:**
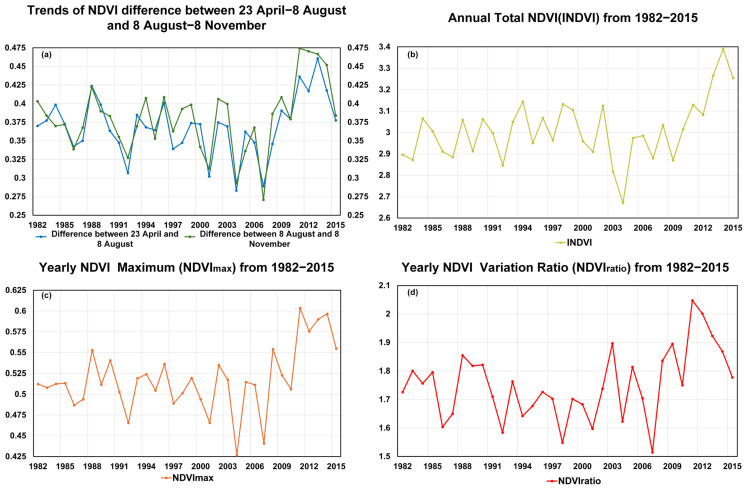
(**a**) The difference in the NDVI between 23 April and 8 August, as well as between 8 August and 8 November, from 1982 to 2015, (**b**) Trends in INDVI from 1982 to 2015, (**c**) Trends in ${NDVI}_{max}$ from 1982 to 2015. (**d**) Trends in *NDVIratio* from 1982 to 2015.

To further evaluate vegetation greenness variations in the HLB, we calculated NDVI differences between 8 August and 23 April and between 8 August and 8 November, assuming that 8 August is the POS across the study period to separate the greening and browning period, as the POS has never been later than this date. As shown in Figure 3a, the difference in the NDVI between the beginning of the greening period and the peak is lower (insignificant but observable) than that between the peak and the end of the browning period, suggesting a sharper decline in autumn in more recent years over the study period. The unusually small difference in 2007 may be due to delayed green-up from low spring temperatures or earlier browning caused by early autumn cooling. The interannual variation in the INDVI shown in Figure 3b indicates a significant upward trend since 2011, suggesting a possible enhancement in regional vegetation productivity driven by climate warming and increased humidity or the implementation of ecological protection measures. However, there are also notable anomalies, such as significant declines in the INDVI in 2003 and 2004, which may be attributed to extreme drought events or disturbances caused by human activity. Figure 3c shows that, during 2004–2015, *NDVImax* displayed pronounced interannual variability, even exceeding that of the INDVI, indicating notable fluctuations in vegetation growth, with marked enhancement after 2010. In Figure 3d, the *NDVIratio*, calculated from *NDVImax*, *NDVImin*, and *NDVIavg*, exhibits a general upward tendency from 2000 to 2015, with pronounced peaks in 2003 and 2011 corresponding to drought years, highlighting the high sensitivity of vegetation to water stress. It should be noted that this upward tendency reflects the overall pattern and does not imply formal statistical significance. Over the long term, the range of *NDVIratio* variations widened during 2000–2015, suggesting that climate change may have increased the instability of vegetation greenness.

### 2.2. Long-Term Trends in Greening and Browning Speeds

**Figure 4:**
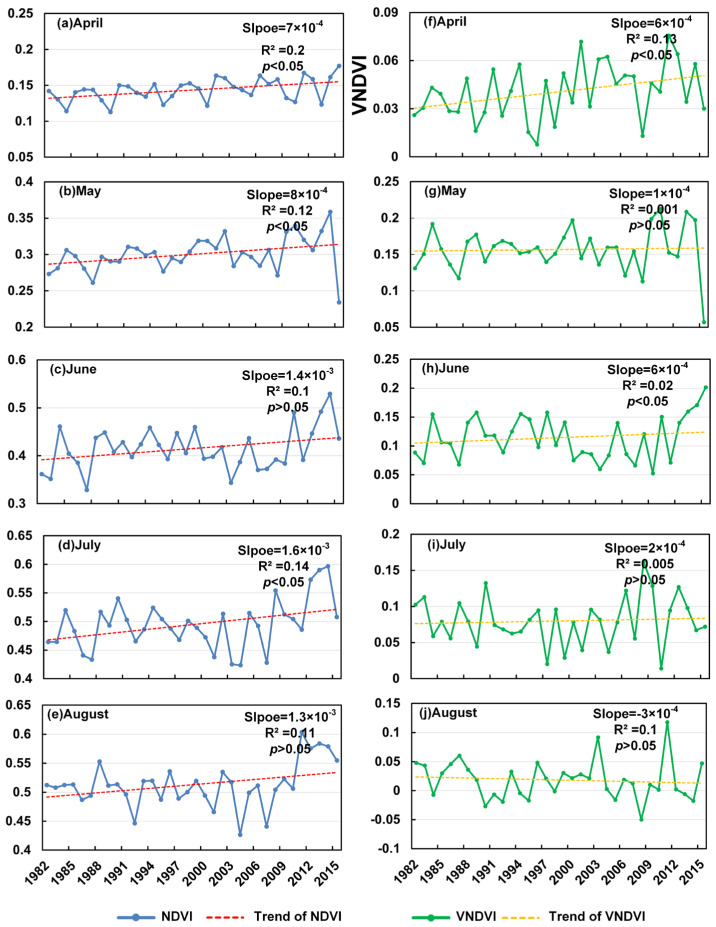
Trends in NDVI in the greening period from 1982 to 2015 (**a**–**e**) and trends in VNDVI in the greening period from 1982 to 2015 (**f**–**j**).

Across the spring greening period (April–August; Figure 4), the NDVI exhibits a general upward trajectory, although the monthly behaviors differ substantially. In April, the NDVI increases at a rate of 7.0 × 10^−4^ yr^−1^ (R^2^ = 0.20, *p* < 0.05), accompanied by a significant rise in the VNDVI (6.0 × 10^−4^ yr^−1^, R^2^ = 0.13, *p* < 0.05). These concurrent positive trends indicate the acceleration of early-spring greening. In May, the NDVI continues to rise significantly (8.0 × 10^−4^ yr^−1^, R^2^ = 0.12, *p* < 0.05), whereas the VNDVI shows an almost negligible change (1.0 × 10^−4^ yr^−1^, R^2^ ≈ 0.001, *p* > 0.05), implying that the greenness enhancement in May primarily reflects accumulated vegetation growth rather than a sustained increase in the greening speed. In June, the NDVI increases moderately (1.4 × 10^−3^ yr^−1^, R^2^ = 0.10, *p* > 0.05) but without statistical significance, while the VNDVI exhibits a significant positive trend (6.0 × 10^−4^ yr^−1^, R^2^ = 0.02, *p* < 0.05), suggesting that early-summer vegetation activity has intensified, even when the mean NDVI changes remain less pronounced. July shows the strongest NDVI increase (1.6 × 10^−3^ yr^−1^, R^2^ = 0.14, *p* < 0.05), yet the VNDVI rises only slightly and insignificantly (2.0 × 10^−4^ yr^−1^, R^2^ ≈ 0.005, *p* > 0.05), consistent with the NDVI approaching its seasonal maximum and the greening rate leveling off. In August, the NDVI maintains a weak positive but non-significant trend (1.3 × 10^−3^ yr^−1^, R^2^ = 0.11, *p* > 0.05), while the VNDVI turns negative (−3.0 × 10^−4^ yr^−1^, R^2^ = 0.10, *p* > 0.05), indicating a decline in late-summer greening acceleration, possibly driven by climatic stress or anthropogenic disturbances. Collectively, Figure 4 highlights the progressive advancement and acceleration of greening in early spring, followed by diminishing growth acceleration toward late summer, marking a gradual transition from rapid vegetative expansion to seasonal stabilization.

**Figure 5:**
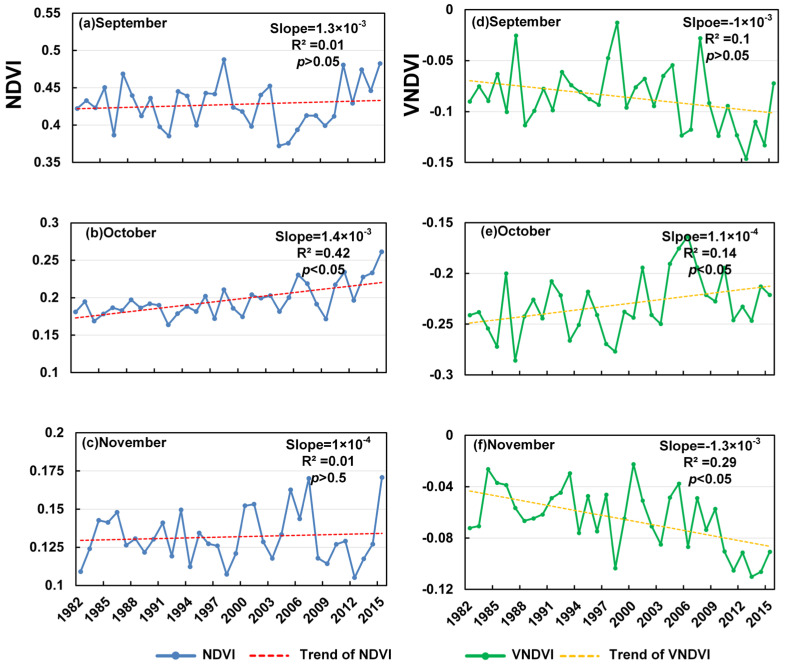
Trends in NDVI in the browning period from 1982 to 2015 (**a**–**c**) and trends in VNDVI in the browning period from 1982 to 2015 (**d**–**f**).

The autumn browning period (September–November; Figure 5) presents more heterogeneous and contrasting temporal patterns. In September, the NDVI shows a weak positive slope (1.3 × 10^−3^ yr^−1^, R^2^ = 0.01, *p* > 0.05), whereas the VNDVI decreases (−1.0 × 10^−3^ yr^−1^, R^2^ = 0.10, *p* > 0.05), suggesting a faster month-to-month increase in browning intensity despite limited change in the mean NDVI. October stands out as an exception: both the NDVI (1.4 × 10^−3^ yr^−1^, R^2^ = 0.42, *p* < 0.05) and VNDVI (1.1 × 10^−3^ yr^−1^, R^2^ = 0.14, *p* < 0.05) increase significantly, implying a temporary slowdown or delay in autumn browning, which provides robust evidence for an extended growing season. In November, the NDVI remains nearly unchanged (1.0 × 10^−4^ yr^−1^, R^2^ = 0.01, *p* > 0.05), while the VNDVI declines sharply (−1.3 × 10^−3^ yr^−1^, R^2^ = 0.29, *p* < 0.05), indicating an accelerated browning rate. However, the divergence between the stable NDVI and markedly negative VNDVI suggests that non-vegetation influences—such as snow accumulation or surface freezing—likely dominate the November signal, rather than continued vegetation senescence. Overall, Figure 5 reveals a pattern of accelerated early-autumn browning in September, a notable slowdown and delay in browning in October, and the apparent intensification of surface browning in November that is probably confounded by cryospheric effects rather than true vegetation change.

### 2.3. Spatial Patterns of the Relationships Between Vegetation Dynamics and Climatic Drivers

**Figure 6:**
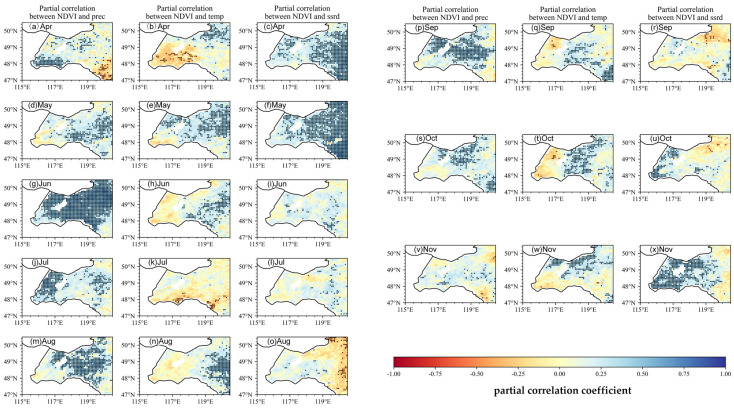
Spatial patterns of partial correlation coefficient between monthly NDVI and preseason climate factors (precipitation P, temperature T, and solar radiation SR). When calculating the partial correlation of the NDVI versus one climate factor (e.g., T), the other two climate factors (e.g., P, SR) are statistically controlled for. Black dots represent pixels with statistical significance (*p* < 0.05), indicating areas where the results are statistically robust.

**Figure 7:**
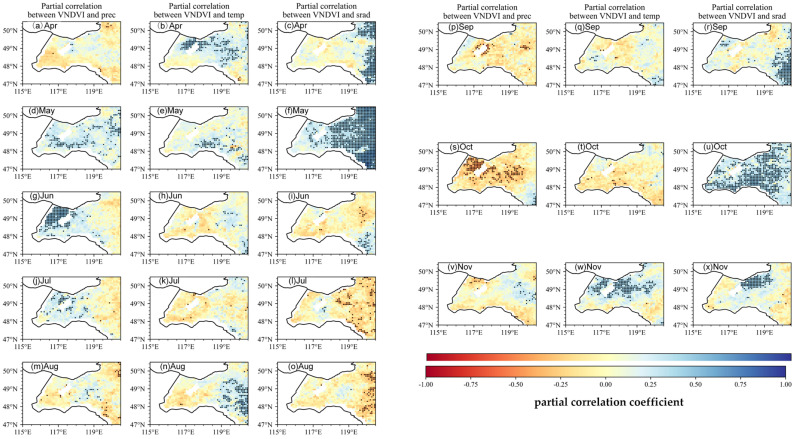
Spatial patterns of partial correlation coefficient between monthly VNDVI and the three climate factors (precipitation P, temperature T, and solar radiation SR). When calculating the partial correlation of the VNDVI versus one climate factor (e.g., T), the other two climate factors (e.g., P, SR) are statistically controlled for. Black dots represent pixels with statistical significance (*p* < 0.05), indicating areas where the results are statistically robust.

Figure 6 and Figure 7 present the spatial distribution of the partial correlation coefficients between the NDVI and temperature (T), precipitation (P), and solar radiation (SR) from April to November. In this study, the temperature, precipitation, and solar radiation are selected as the primary climatic drivers due to their significant influences on vegetation growth and productivity. The temperature plays a crucial role in regulating plant metabolism, photosynthesis, and overall ecosystem productivity. It is widely acknowledged as a key factor in vegetation–climate interactions [23]. Precipitation directly impacts water availability, which is vital for vegetation growth, especially in semi-arid and arid regions, where water stress is a limiting factor [24]. Solar radiation is considered as it is essential for photosynthesis and plant development. The combined effects of these three factors—the temperature, precipitation, and solar radiation—have been well documented in the literature as fundamental drivers of vegetation dynamics and productivity across diverse ecosystems [25,26]. Significance is encoded by the color in the maps: bluer tones indicate more significant relationships, whereas redder tones indicate weaker or non-significant ones. These maps reveal how vegetation responds to climatic forcing at successive stages of the growing season, as well as spatiotemporal heterogeneity in these responses.

At the basin scale, the temperature exerts a clear positive influence on the spring NDVI: during the spring greening period, many pixels in the grassland zone exhibit a partial r = 0.35 for the NDVI–T relationship in May–June, with numerous pixels reaching statistical significance (*p* < 0.05). Early-season precipitation is also highly influential in the grassland zone: the NDVI–P partial r typically equals 0.50 in May and reaches 0.60 in June, underscoring the critical role of water availability during canopy recovery. By contrast, NDVI–SR correlations are weak in April but become positive in May over parts of the forest zone and adjacent grassland, with typical values = 0.30. As the season progresses into midsummer, the NDVI–temperature association weakens and, in places, reverses sign. During July–August, several areas within the grassland zone show negative partial r values = −0.30, consistent with the heat stress suppression of greenness in these open ecosystems. The NDVI nevertheless remains sensitive to precipitation throughout this period (peak NDVI–P = 0.60 in June, with values = 0.40–0.50 persisting into July in the grassland zone), while localized negative NDVI–P patches (r = −0.25) emerge in July–August in some low-lying or poorly drained grassland subregions. NDVI–SR shifts from positive in June (r = 0.40) to weakly or locally negative in July–August (local minima r = −0.45) toward the forest zone margin, implying that high radiation combined with heat can suppress greenness along the eastern margin.

During the autumn browning period (September–November), climatic controls shift between the two ecological zones. In September, the NDVI commonly shows a positive partial r with the temperature and radiation (r = 0.35) across parts of both zones, while the influence of precipitation remains generally weak. October becomes precipitation-dominated at the basin scale: NDVI–P partial r ≈ 0.45 across much of the grassland zone, with many pixels statistically significant, whereas temperature and radiation influences decline. By November, the temperature regains prominence—particularly within the forest zone, where the NDVI–T partial r is near 0.55—and precipitation effects become negligible. Overall, the NDVI maps reveal seasonal shifts in the dominant climatic drivers: the temperature and precipitation jointly control spring green-up in the grassland zone; precipitation dominates mid-summer NDVI patterns (especially in grassland areas); and the temperature, with local radiation effects, becomes increasingly important during autumn senescence—most notably within the forest zone.

Figure 7 shows that the VNDVI captures even strong and spatially explicit sensitivities of growth rates to climatic variability across the two zones. In the spring greening period (May–June), representative VNDVI–T partial r values in the grassland zone are = 0.50 (May) and = 0.45 (June), indicating that moderate warming substantially accelerates canopy expansion in open grasslands. VNDVI–P correlations are similarly strong in spring in the grassland zone (r = 0.50), while VNDVI–SR becomes notable in June (r = 0.40), particularly near the forest–grassland interface, reflecting the synergistic effects of light and moisture during rapid greening. In summer (July–August), the sign and magnitude of the VNDVI correlations shift markedly and vary by zone. VNDVI–T turns negative across parts of the grassland zone and the eastern margin, with representative r = −0.40, consistent with heat suppression of growth rates. Sensitivity to precipitation weakens in July and becomes negative in August in the grassland zone (r = −0.30), compatible with waterlogging or excess moisture effects in some subregions. The VNDVI–SR relationship changes from weakly positive in June to clearly negative in July–August toward the forest margin (r = −0.45), suggesting that the combined stresses of high radiation and temperatures reduce sustained growth here.

During the autumn browning period (September–November), the VNDVI again exhibits distinct month-to-month shifts across zones. VNDVI–T is weakly negative in September and clearly negative in October with representative r = −0.40, particularly within the grassland zone, indicating faster browning under warmer autumn conditions. The VNDVI–P relation changes from modestly positive in September to negative in October (r = −0.35) and becomes near-neutral by November. Solar radiation has a limited influence in early autumn but becomes moderately positive in October–November (r = 0.35), an effect that is relatively more apparent in the forest zone, where the canopy structure modulates light regimes. Spatially, the strongest temperature–VNDVI associations occur in the grassland zone (heightened thermal sensitivity), whereas portions of the forest zone display negative temperature–VNDVI correlations in autumn, consistent with frost-related premature senescence. Overall, the VNDVI reveals larger effect sizes and sharper sign reversals than the NDVI, documenting the positive climatic control of growth rates in spring, negative impacts of midsummer heat and excess moisture, and a late-season transition toward radiation (and, locally, temperature) control—patterns that a static greenness index alone would underrepresent.

### 2.4. Divergent Responses of Greenness Versus Greening and Browning Speeds to Climate

**Figure 8:**
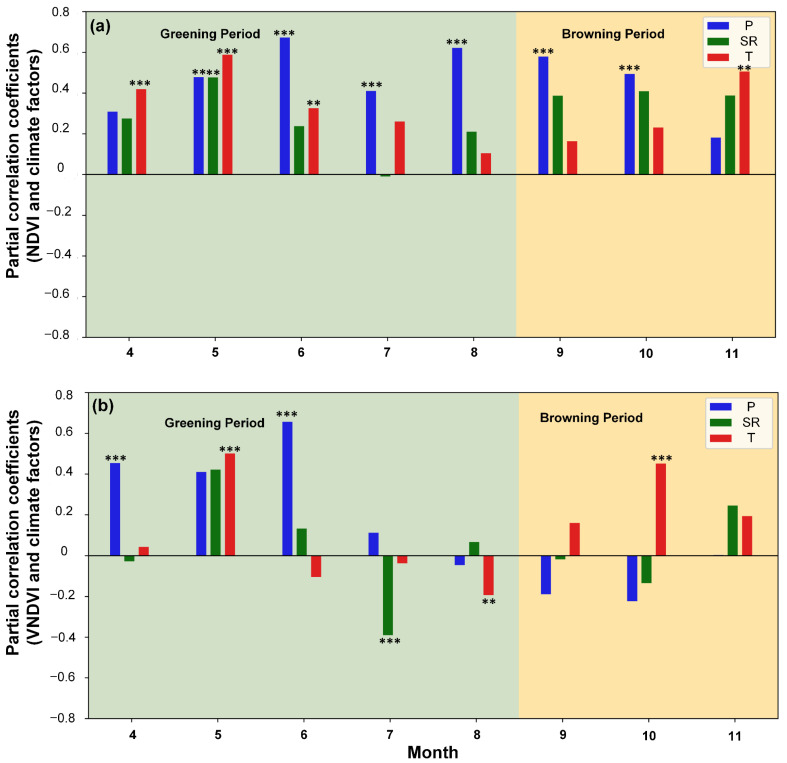
(**a**) Partial correlations between NDVI and climatic drivers (precipitation P, temperature T, and solar radiation SR) across the entire study area, (**b**) Partial correlations between VNDVI and climatic drivers. Double asterisks (**) and triple asterisks (***) denote significance at *p* < 0.05 and *p* < 0.01, respectively.

To isolate the independent effects of individual climatic drivers on vegetation dynamics, the temporal patterns of the partial correlation coefficients between the NDVI, the VNDVI, and the three climatic drivers of the entire study area from April to November are shown in Figure 8. The NDVI was mainly driven by the temperature in April (r = 0.3) and significantly correlated with all three factors in May (P = 0.49, SR = 0.48, T = 0.56), reflecting their synergistic roles in promoting canopy greening. In June, the NDVI exhibited its strongest partial correlation with precipitation across the year (r = 0.65), underscoring the critical role of the water supply in maintaining leaf greenness, whereas the effects of the temperature and radiation slightly weakened (both around 0.20–0.30). In July, precipitation remained the primary driver (r = 0.37), with solar radiation turning weakly negative (r = −0.06) and the temperature maintaining a moderate positive influence (r = 0.21). By August, precipitation continued to dominate (r = 0.64), while the contributions of the temperature and radiation were small (both ≤0.15). During the autumn browning stage (September–November), the dominance of precipitation regarding the NDVI became weaker overall, while the contributions of the temperature and radiation gradually increased; the temperature became the main driver in November (r = 0.44). These temporal transitions are consistent with a cumulative canopy perspective for greenness (Figure 8a). Overall, the NDVI’s temporal responses exhibited distinct seasonal transitions—canopy greening in spring jointly driven by the temperature and radiation, precipitation exerting the strongest control in summer, and regulation progressively shifting back toward the temperature in autumn.

The strong influence of precipitation on the VNDVI (Figure 8b), combined with the dominance of temperature regarding the NDVI (Figure 8a), further indicates that changes in the NDVI are, in early spring, closely related to thermal release and moisture support rather than structural growth alone. The VNDVI was significantly and positively correlated with the temperature in May (r = 0.48, *p* < 0.01) and with precipitation in June (r = 0.65, *p* < 0.01), consistent with the NDVI and confirming the importance of sufficient water for leaf expansion. From May to July, the positive influence of solar radiation weakened and became negative, reaching -0.38 in July, reflecting entry into a stable growth stage in which greening rates are less responsive. In August, the VNDVI exhibited negative correlations with precipitation in several areas (r = −0.08), likely due to waterlogging, while a negative correlation with the temperature also emerged (r = −0.23, *p* < 0.05), highlighting the heat stress suppression of the greening rate. During the autumn browning stage, the VNDVI remained weakly negative with precipitation and radiation in September–October but became increasingly positive with the temperature, peaking in October (r = 0.44, *p* < 0.01). By November, precipitation’s effect was minimal (near zero), while the temperature and radiation showed modest positive effects. Relative to the NDVI, these patterns show that the VNDVI captures accelerated greening in May and the rapid turnover of dominant drivers into summer, and the VNDVI reveals growth suppression under combined heat and excess moisture that can be masked by a persistently high NDVI (Figure 8b). In late autumn, the VNDVI also highlights the radiation-associated mitigation of declines (e.g., strong VNDVI–SR associations in October–November), consistent with the rate-based interpretation of growing season extension. Spatially, late-summer declines in the VNDVI coincide with known grazing zones, indicated by Figure 4 and Figure 7, suggesting the modulation of process rates by human activity rather than changes in static greenness alone. Taken together, the joint use of the NDVI and VNDVI improves the detection of seasonal driver transitions and facilitates the interpretation of ecosystem carbon flux seasonality. Considering that the VNDVI values have become negative since September, these patterns suggest that, even though the water supply helps to maintain greenness (Figure 8a), intensified warming accelerates the browning rate in the late season (October–November; Figure 8b).

## 3. Discussion

### 3.1. Interpretation of Key Temporal Trends in Vegetation Growth Speeds

Over more than 30 years, as demonstrated in Figure 1, Figure 2, Figure 3, Figure 4 and Figure 5, vegetation in the HLB exhibited significant temporal changes in vegetation growth speeds, characterized by accelerated spring greening rates, constrained summer growth, and delayed autumn browning [27]. These trends not only reflect the general phenomena under climate warming but also highlight the distinctive response patterns of the HLB as a typical ecotone. Specifically, vegetation processes in different seasons are regulated by heterogeneous climatic drivers and exhibit pronounced spatial variability.

The significant increase in the VNDVI from April to May indicates the marked acceleration of spring canopy development, consistent with the globally observed phenomenon of advanced green-up under climate warming [28,29]. This is primarily attributed to earlier snowmelt (reducing red-band reflectance) and the relaxation of thermal growth constraints [30]. In particular, the strongest correlations between the temperature and VNDVI were observed over the southwestern grassland zone of the basin, suggesting that these steppe ecosystems are highly sensitive to thermal variability. In contrast to the pronounced spring trend, the VNDVI during the peak summer months showed no significant change, reflecting physiological saturation or moisture/nutrient limitations that constrain further vegetation growth at this stage. Such a pattern aligns with evidence from temperate forest ecosystems, where the carbon sink capacity becomes limited in summer [31]. In other words, although the NDVI remained at relatively high levels, the actual growth rate of vegetation had slowed or plateaued. Notably, the decline in the browning speed observed in October indicates a delay in vegetation senescence (Figure 5), which may be associated with autumn warming and extended favorable late-season conditions [32]. Such an extension of the growing season not only prolongs the photosynthetically active period but also carries important implications for regional carbon cycling. However, the spatial variation in the NDVI trends (Figure 5) shows that greening acceleration was strongest in the central and southwestern areas, which are now considered part of the grassland zone, and weaker in the forest zone, located in the eastern part of the basin (Great Khingan foothills). These patterns reflect ecosystem-specific differences in vegetation dynamics within the basin’s grassland–wetland–forest ecotone.

### 3.2. Dominant Climatic Drivers and Seasonal Shifts

The partial correlation analysis (Figure 8) further proves that the dominances of the temperature, precipitation, and radiation are highly varied in different seasons from the perspective of the VNDVI [33]. The VNDVI is generally more sensitive than the NDVI to these controls, reflecting its sensitivity to month-to-month changes in vegetation activity.

Temperature is the primary driver of VNDVI variability, with radiation playing a secondary role (Figure 8) [34]. This contrasts with semi-arid regions, where spring vegetation growth is typically constrained by precipitation, suggesting that the HLB is more sensitive to temperature anomalies during the snowmelt transition period [35]. During the spring greening period, the temperature is the principal correlate of the VNDVI in much of the grassland zone, with representative partial r values of around 0.50 in May and 0.45 in June. Precipitation also shows strong positive partial correlations with the VNDVI in spring, underscoring the joint importance of thermal and moisture conditions for the rate of canopy development (Figure 6 and Figure 7). In summer, the spatial patterns change: a high temperature correlates negatively with the VNDVI in large portions of the grassland zone [36]. These patterns suggest that both thermal stress and hydrological conditions constrain growth rates during the warm season. The positive effect of radiation becomes more pronounced, maintaining photosynthetic activity and delaying senescence induced by declining temperatures. This mechanism is often underestimated in NDVI-based studies. Concurrently, in the northeastern part of the basin, the correlation between the temperature and VNDVI becomes significantly negative, likely reflecting premature leaf senescence caused by low-temperature frosts. Together, these autumn patterns indicate a shift in climatic correlates as radiation becomes more relevant in October–November, while temperature effects vary spatially.

These results demonstrate that vegetation in the HLB is controlled by different climatic factors across seasons, i.e., the temperature in spring, precipitation in summer, and radiation in autumn. Furthermore, the VNDVI provides a clearer depiction of climate-driven differences at the “rate” level. For instance, the NDVI may only indicate an overall greenness increase, while the VNDVI reveals how climatic factors differentially regulate vegetation growth and senescence rates across seasons and ecosystems [37].

### 3.3. Implications for Ecosystem Functioning

The findings above indicate that the vegetation dynamics in the HLB are not only seasonally regulated by distinct climatic drivers but also shaped by spatially heterogeneous responses across regions with different ecosystems [38]. Such variability inevitably influences ecosystem functioning, particularly in terms of carbon cycling, ecological vulnerability, and potential climate feedbacks.

The accelerated spring rate of growth (as indicated by the VNDVI) suggests the potential for increased early-season carbon uptake, whereas reduced summer growth rates under heat or moisture stress could partially offset these gains. Direct quantification of carbon flux changes, however, requires independent flux data. The radiation-driven delay in autumn senescence further prolongs the active photosynthetic period, potentially extending the duration of carbon sequestration and enhancing the regional carbon sink capacity. This seasonal dynamic supports the “browning speed buffer” concept proposed by Meng et al. [39], highlighting how temporal variations in vegetation growth rates modulate carbon fluxes.

From an ecological vulnerability perspective, grasslands, particularly in the central and southwestern areas (now classified as the grassland zone), are sensitive to summer drought and heat stress, as evidenced by the pronounced decline in the VNDVI in these regions (Figure 7) [40]. Such responses align with observed trends of grassland degradation in the HLB [41], suggesting that these ecosystems may face intensified risks under future climate variability. In contrast, forests in the eastern part (forest zone) exhibit weaker responses to temperature anomalies compared to the grassland zone (Figure 7), indicating greater resilience in terms of sustaining growth under stress conditions. Nevertheless, the significant sensitivity to autumn frost in these forests poses a potential risk to late-season productivity and leaf retention, which could influence carbon and energy fluxes.

These phenological shifts also have important implications for regional climate feedback [42]. Extended active periods could influence regional water and energy fluxes (e.g., evapotranspiration), but assessing net impacts on regional hydroclimates requires coupled ecohydrological or flux-based analyses beyond the scope of this study [43]. At the same time, enhanced vegetation cover can alter the surface energy balance by modulating the surface albedo, thereby affecting local radiation budgets and introducing complex bio-geophysical feedbacks. The interplay of these processes highlights the necessity of considering both carbon–climate interactions and ecosystem-specific responses when assessing the impacts of climate variability on regional vegetation dynamics.

### 3.4. Complementary Insights from VNDVI vs. NDVI and Implications

The VNDVI and NDVI capture fundamentally distinct aspects of vegetation dynamics, providing complementary yet divergent ecological information [44]. The comparative analysis (Figure 4, Figure 5, Figure 6, Figure 7 and Figure 8) highlights this, with the VNDVI offering process-level insights that the NDVI is unable to resolve [45]. In terms of temporal sensitivity, the NDVI primarily reflects the cumulative canopy status, such as the gradually increased and observed peak values in July or August, but this integrative signal often obscures transient physiological transitions. By contrast, the VNDVI explicitly quantifies the rates of change in canopy development, enabling the detection of accelerated greening in May and slowed senescence in October (Figure 5 and Figure 6). These findings reveal critical phenological shifts that are invisible to the NDVI (Figure 1), thereby explaining why NDVI-based studies have systematically overlooked the compressed spring recovery period in the HLB (Figure 3a). This demonstrates that the VNDVI is not merely an alternative indicator but a necessary complement in capturing short-term phenological transitions that govern seasonal carbon fluxes.

In terms of process-specific climate responses, the VNDVI provides mechanistic clarity that the NDVI lacks [46]. While the NDVI showed a broad significant correlation with the temperature in spring, the VNDVI further revealed how warming accelerated growth rates at the beginning while it lost dominance soon in June, suggesting fast-changing climate drivers for vegetation growth rates [47]. In summer, persistently high NDVI values masked growth suppression with significant positive correlations, whereas the VNDVI revealed insignificant but varying positive-to-negative relationships with precipitation (Figure 8). In autumn, while the NDVI suggested persistent greenness, the VNDVI identified the dominant role of radiation in delaying senescence, thereby elucidating the underlying drivers of growing season extensions. These results emphasize that the VNDVI more effectively links phenological processes with their specific climatic controls.

Beyond climate influences, the VNDVI also proves to be potentially responsive to anthropogenic disturbances [48]. The declines in the VNDVI during August and September (Figure 3 and Figure 4) correspond spatially with grazing zones, indicating that human activity also strongly affects grass growth, which needs further investigation with additional datasets [27]. Such sensitivity is critical because the NDVI, as a cumulative index, is largely insensitive to rapid disturbances such as mowing-induced senescence or grazing pressure. Thus, the VNDVI provides a more robust framework for disentangling natural climatic variability from human-induced impacts on vegetation dynamics. This distinction has important implications for both ecological monitoring and land management, as it highlights the necessity of integrating rate-based indicators into future assessments of ecosystem vulnerability and resilience under global change.

These findings carry important methodological and ecological implications [49]. The VNDVI’s close linkage to canopy development kinetics allows it to more accurately capture short-term physiological processes that drive ecosystem carbon fluxes [50]. For example, the accelerated spring greening detected by the VNDVI provides a better predictor of gross primary productivity (GPP) variations compared to the NDVI [6]. Similarly, the slower browning detected in autumn (VNDVI > NDVI) may indicate prolonged respiration periods, which are critical for net carbon budgeting. In addition, the VNDVI helps to partially alleviate scale mismatches that often limit remote sensing applications. While the coarse-resolution NDVI (8 km) masked important wetland–grassland contrasts in climate responses, the VNDVI’s focus on process dynamics partially resolved these differences by isolating functional vegetation responses. Taken together, these insights underline the utility of integrating the VNDVI alongside the NDVI in ecosystem monitoring, as this dual approach improves the detection of climate impacts, enhances carbon flux assessments, and strengthens the capacity for proactive ecological management.

Nevertheless, although this study primarily utilized the GIMMS NDVI3g dataset, which offers the advantage of a long-term time series, its relatively coarse spatial resolution still limits the ability to capture fine-scale vegetation changes within smaller sub-regions of the HLB. Additionally, the land cover types in this study were not differentiated in detail, which may have masked certain ecosystem-specific responses. Furthermore, this research focused primarily on the relationship between climatic factors and vegetation change rates (VNDVI), without systematically accounting for the influence of human activities. Future studies could incorporate datasets related to land use, socioeconomic development, and anthropogenic disturbances to better understand the coupled impacts of climate change and human activity on the terrestrial vegetation dynamics in the region, aiming to provide a more comprehensive and reliable explanation.

## 4. Materials and Methods

### 4.1. Study Area

**Figure 9:**
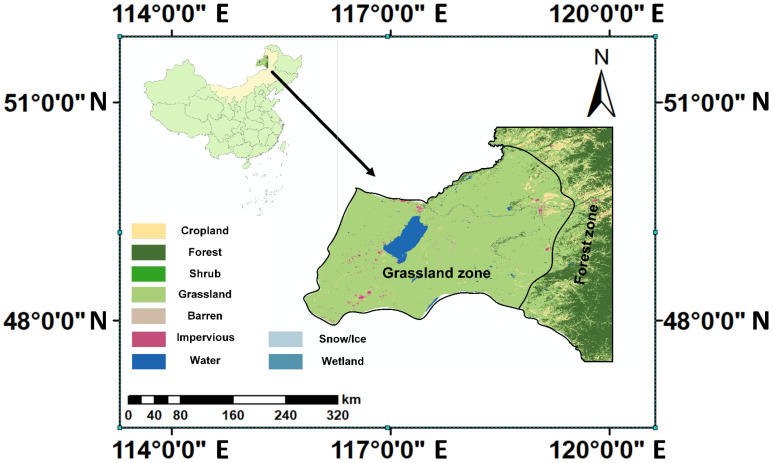
Geographical location of the Hulun Lake Basin in Northeastern Inner Mongolia, China.

The HLB is situated in the Eastern Mongolian Plateau, spanning from 46°58′28″ to 49°59′57″ N and 112°18′16″ to 122°26′45″ E, covering a total area of around 118,835 km^2^. In this study, we mainly focus on the transition area of grassland–cropland–woodland in the basin, covering a region of 84,530 km^2^ (Figure 9). The topography of the basin transitions from the Greater Khingan Mountains in the east to the Hulunbuir grasslands and the up-per reaches of the Kherlen River in the west, with generally gentle terrain and elevation ranging from 444 m a.s.l to 1707 m a.s.l [51]. As mentioned by Zhou, the HLB is characterized by a mid-temperate continental steppe climate, with the mean annual precipitation ranging from approximately 250 to 360 mm and a basin-wide average of about 356 mm. Precipitation is predominantly concentrated in summer, and a distinct temperature gradient—decreasing from south to north—reflects the influence of altitudinal and ecological zonation [52].

Administratively, the basin covers several regions within Hulunbuir City, Inner Mongolia Autonomous Region, China, including Hailar District, Manzhouli City, Yakeshi City, and the New/Old Barag Banners, and extends into parts of Dornod and Sukhbaatar Provinces in Mongolia. The region is hydrologically rich, with major rivers including the Kherlen, Hailar, Halaha, and Orkhon Rivers. Prominent lakes in the basin include Hulun Lake, Beier Lake, and Wulannuoer Lake. Hulun Lake, with a surface area of approximately 2036.6 km^2^, is the third-largest inland lake on the Mongolian Plateau.

The basin contains diverse land cover types, including cultivated land, grassland, forests, shrubland, wetland, water bodies, artificial surfaces, and sparsely vegetated land. This area can be broadly divided into two ecological regions: the southwestern grassland zone and the eastern forest zone. In the grassland zone, temperate steppe communities are typically constrained by moisture availability; vegetation activity therefore responds sensitively to intra-seasonal fluctuations in water supply and temperature. Moderate warming combined with sufficient precipitation generally promotes early-season growth, whereas excessive heat and soil moisture deficits can suppress mid- to late-season greenness and accelerate senescence [51,52]. In the forest zone along the foothills of the Greater Khingan Mountains, cold–temperate coniferous and mixed forests (dominated by Larix gmelinii, Betula platyphylla, and Pinus sylvestris) are primarily shaped by thermal constraints and frost risks. Accumulated warmth facilitates spring leaf-out and extends the growing season, whereas cool summers or frost risks in September can delay phenological development and restrict peak canopy growth [53]. This spatial framework underpins the heterogeneous climate–vegetation relationships analyzed in the following sections. The HLB can thus be described as a grassland–wetland–forest ecotone, i.e., a mosaic transition zone featuring sharp land cover juxtapositions characterized by continuous gradients of moisture and temperature associated with west–east and south–north contrasts and with elevational differences in the eastern uplands. Climatically, the region has a temperate, semi-arid continental–monsoon regime governed by the mid-latitude westerlies and the East Asian summer monsoon: summers are warm and wet; winters are long, cold, and dry; and approximately 75–85% of the annual precipitation falls during May–September [54]. Over recent decades, statistically significant warming and heightened hydroclimatic variability, including the redistribution of seasonal precipitation, have been documented in the HLB and the broader Mongolian Plateau, with direct implications for vegetation phenology, biomass accumulation, and the lake–wetland water balance [55]. These varied landscapes provide a complex environmental backdrop for the analysis of vegetation phenology and biomass dynamics. To enhance ecological protection in the border region, the Chinese government established the Hulun Lake Nature Reserve in 1986. In 1994, under a trilateral agreement among China, Mongolia, and Russia, the Hulun Lake Nature Reserve, Mongolia’s Daurian Nature Reserve, and Russia’s Daursky Nature Reserve were jointly designated as the China–Mongolia–Russia Daurian International Nature Reserve. In 2002, the Hulun Lake National Nature Reserve was listed as a Ramsar Site and incorporated into UNESCO’s World Biosphere Reserve Network.

### 4.2. Datasets

The NDVI is one of the most widely used indicators in assessing vegetation dynamics and is recognized as a reliable proxy for the monitoring of terrestrial vegetation growth. In this study, we used the GIMMS NDVI3g dataset [56], covering the period from 1982 to 2015 (as the study period). The dataset has been systematically corrected for orbital drift, sensor calibration, viewing geometry, and volcanic aerosols [57], ensuring data continuity and stability. It provides biweekly NDVI observations with a spatial resolution of 0.083° (approximately 8 km), making it well suited for the long-term monitoring and analysis of vegetation variation in the HLB over more than three decades. The selection of the GIMMS NDVI3g dataset was based on its demonstrated reliability and consistency in capturing interannual vegetation variability. Previous evaluation studies have shown that GIMMS NDVI3g maintains strong agreement with other NDVI products, such as VIP15 NDVI and earlier GIMMS versions, in representing vegetation greenness dynamics across various ecosystems [58]. This high consistency and long temporal coverage make GIMMS NDVI3g particularly suitable for multi-decadal analyses of vegetation change and its climatic drivers. A phenology camera has been installed on a tower in the central HLB near Kherlen River since March of 2024, allowing us to obtain the actual grassland phenological status of the HLB.

Climatic drivers are crucial external factors affecting vegetation dynamics. In this study, we employed a high-resolution meteorological forcing dataset for China, developed and released by the National Tibetan Plateau Data Center [59], which includes key variables such as the air temperature, precipitation, and radiation. We used the meteorological dataset covering the period from 1982 to 2015, with a temporal resolution of 3 h and a spatial resolution of 0.1°. It integrates surface meteorological observations from the China Meteorological Administration (CMA), reanalysis data sources such as the Princeton Global Meteorological Forcing Dataset (Princeton) and the Global Land Data Assimilation System (GLDAS), and satellite-based products such as the Tropical Rainfall Measuring Mission (TRMM). All data have undergone rigorous assimilation and quality control. To ensure spatial consistency with the NDVI data, all meteorological variables were resampled to a spatial resolution of 0.083°, providing essential climatic inputs for the analysis of the drivers of vegetation phenology and biomass variation in the study area. In our study, we mainly use three climatic drivers—precipitation, temperature and solar radiation—to illustrate the relationships between the indices, namely the NDVI, VNDVI, and climate factors.

### 4.3. Data Processing and Analysis

Four indices derived from the biweekly NDVI, namely the INDVI, VNDVI, ${NDVI}_{max}$, and *NDVI_ratio_*, were calculated and analyzed (Table 1), while the main focus was the comparison of the NDVI with the VNDVI. In Table 1, these four commonly used NDVI-derived indices are summarized, each with its biological significance. The annual total NDVI (INDVI) represents the sum or integral of the NDVI values over the entire growing season, providing a proxy for the overall biomass or productivity within a given year. This index has been widely used in assessing annual vegetation productivity and biomass [60]. The NDVI change rate (NDVI change rate, including greening and browning rates) measures the rate of change in the NDVI between consecutive periods, which is especially useful in capturing the speed of vegetation greening or browning. This index helps to quantify vegetation dynamics in response to environmental disturbances or seasonal variations [61]. The annual maximum NDVI (${NDVI}_{max}$) indicates the peak vegetation greenness during the growing season and is often used as a surrogate for potential productivity [62]. Finally, the variation ratio of the annual NDVI (*NDVIratio*) quantifies the annual variation in the NDVI by calculating the difference between the maximum and minimum NDVI values, normalized by the total annual NDVI. This index is essential in assessing seasonal fluctuations and the overall vegetation response to climate variability [63].

(1) $$INDVI=\sum {NDVI}_{biweekly}$$

(2) $${VNDVI}_{t}={NDVI}_{t}-{NDVI}_{t-1}$$

(3) $${NDVI}_{ratio}=\frac{{NDVI}_{max}-{NDVI}_{min}}{{NDVI}_{avg}}$$

where *t* is the time, and *NDVI_t_* and *NDVI_t__−_*_1_ are the NDVI at time *t* and *t* − 1 (biweekly duration), respectively, for a given year. A positive VNDVI*_t_* represents the speed of canopy development, and a negative *VNDVI_t_* represents the speed of canopy senescence. *NDVImin* and *NDVIavg* are the minimum and annual average NDVI within the growing season of a given year.

First, we analyzed terrestrial vegetation changes in the HLB by examining the NDVI and related indicators to assess the vegetation dynamics over the study period. We calculated greening and browning periods and derived indices such as the INDVI, VNDVI, ${NDVI}_{max}$, and *NDVIratio* to characterize the vegetation phenology in the HLB by using biweekly NDVI time series from 1982 to 2015. Subsequently, since partial correlation analysis allows for the examination of the linear relationship between two variables while controlling for the influences of other variables, we applied this method to determine the associations between climatic factors and the NDVI/VNDVI. This approach excludes the effects of the other two climatic factors from one factor and allows us to observe the influence of a single climatic factor on the NDVI/VNDVI. The analyses were processed with PyCharm Community Edition 2024.1 and Arcgis 10.

## 5. Conclusions

This study investigated long-term trends in vegetation dynamics in the HLB at a biweekly scale using both greenness (NDVI) and phenological process-based indicators (greening/browning speeds, VNDVI). By integrating spatiotemporal trend analysis and climatic driver attribution, we reveal the responses of vegetation to climate change across phenological phases. The results indicate that stronger greening acceleration in spring occurs in central/southwestern grasslands, while weaker responses are found in the transition area from grassland to forest in the east. From the perspective of climatic drivers, climate warming in spring accelerates vegetation green-up, while high temperatures and moisture stress in summer suppress growth rates. In contrast, moderate warming and increased radiation in autumn can effectively delay vegetation browning. Such variability influences ecosystem functioning in the HLB, particularly in terms of carbon cycling, ecological vulnerability, and potential climate feedbacks, highlighting the necessity of considering both carbon–climate interactions and ecosystem-specific responses when assessing the impacts of climate variability on regional vegetation dynamics. Since the VNDVI and NDVI provide complementary yet divergent ecological information, as the NDVI primarily reflects the cumulative canopy status and the VNDVI offers process-level insights, the integrative utilization of the VNDVI alongside the NDVI in ecosystem monitoring could enhance carbon flux assessments and strengthen the capacity for proactive ecological management in this vulnerable transitional zone.

## Figures and Tables

**Table 1:** NDVI-derived indices.

Index	Definition	Measurement Indicator	Biological Significance
INDVI [41]	Annual total NDVI	Overall productivity and biomass	Annual vegetation productivity
VNDVI [42]	NDVI change rate	Vegetation phenology	Vegetation change rate
*NDVImax* [43]	Maximum NDVI within the growing season of a year	Overall productivity and biomass	Annual vegetation productivity
*NDVIratio* [44]	Normalized difference in NDVI within the year	Interannual variability in productivity	Interannual biomass comparison

## Data Availability

The data presented in this study were obtained from the following resources available in the public domain: GIMMS NDVI3g (https://data.nasa.gov/dataset/global-vegetation-greenness-ndvi-from-avhrr-gimms-3g-1981-2022-6b8d3 (accessed on 22 October 2025)) and China Meteorological Forcing Dataset (CMFD) via TPDC (https://www.dess.tsinghua.edu.cn/en/info/1226/2766.htm (accessed on 22 October 2025)).
